# Comparison of Low-Dose Interleukin 2 Therapy in Conjunction With Standard Therapy in Patients With Systemic Lupus Erythematosus vs Rheumatoid Arthritis: A Systematic Review

**DOI:** 10.7759/cureus.56704

**Published:** 2024-03-22

**Authors:** Muhammad Faisal Riaz, Gourav Garg, Lotanna Umeano, Sadaf Iftikhar, Sarah F Alhaddad, Christian N Paulsingh, Pousette Hamid

**Affiliations:** 1 Internal Medicine, California Institute of Behavioral Neurosciences & Psychology, Fairfield, USA; 2 Oncology, Nuclear Medicine, Oncology and Radiotherapy Institute (NORI), Rawalpindi, PAK; 3 Orthopaedics, California Institute of Behavioral Neurosciences & Psychology, Fairfield, USA; 4 Orthopaedics, King's Mill Hospital, Sutton-in-Ashfield, GBR; 5 Pediatrics, California Institute of Behavioral Neurosciences & Psychology, Fairfield, USA; 6 Pathology, St. George's University School of Medicine, St. George's, GRD; 7 Neurology, California Institute of Behavioral Neurosciences & Psychology, Fairfield, USA

**Keywords:** low-dose interleukin 2 therapy, low-dose il2 therapy, immunomodulation, il2 therapy, ra, rheumatoid arthritis, sle, systemic lupus erythematosus

## Abstract

This systematic review aims to compare the efficacy and safety of a novel immunotherapy with low-dose interleukin 2 (IL2) across two of the most prevalent autoimmune diseases i.e. systemic lupus erythematosus (SLE) and rheumatoid arthritis (RA). Contemporary therapeutic practices have not been able to achieve complete remission from these autoimmune disorders. In contrast, low-dose IL2 has shown promise in achieving this therapeutic goal via inducing self-tolerance in patients with autoimmune diseases; however, due to variable irregularities among autoimmune processes of variable diseases, the benefit of low-dose IL2 could not be determined among different autoimmune diseases. Therefore, we conducted a study to compare low-dose IL2 therapy effects on SLE and RA. We systematically screened four databases: PubMed, Medical Literature Analysis and Retrieval System Online (MEDLINE), PubMed Central (PMC), and Google Scholar. Inclusion and exclusion criteria were implemented. Quality appraisal of studies chosen for the review was done using the Cochrane Risk-of-Bias (RoB) assessment tool for randomized controlled trials, and the Newcastle-Ottawa Scale (NOS) and JBI critical appraisal tool for non-randomized clinical trials. Information was gathered from seven articles: three randomized controlled trials and four non-randomized clinical trials. Our review concluded that low-dose IL2 therapy in conjunction with respective standard therapies for SLE and RA has a higher efficacy and safety profile as compared to standard therapy alone and the therapeutic effects were comparable in both SLE and RA patients treated with low-dose IL2; however, this novel intervention does not seem to have a significant corrective effect on the biomarkers of RA as it does for SLE biomarkers.

## Introduction and background

Our immune system is a diverse machinery that fulfills the primary function of protecting us from foreign and infectious agents. This complex system, which fights foreign entities that have invaded the human body, is itself an intricate balance between several components comprising the whole immune system. These components keep the body in a state of tolerance to its own immune system, also called “immunological inertness to self”. Thus, disruption of the balance in this diverse system can lead to pathology that manifests in primarily two different ways; immune deficiency syndromes and autoimmune diseases [1].

Autoimmune diseases are a diverse group of chronic conditions. These conditions usually share common pathway abnormalities accompanied by sometimes subtle, and at other times outright differences among the anomalies in their pathways which lead to distinct clinical manifestations in each autoimmune disease along with a range of common features [2]. These abnormalities in their shared and distinct pathways together lead to inappropriate immune responses directed to self-antigens present in healthy cells in the human body. It leads to the disruption and destruction of normal machinery in our bodies and manifests in various clinical presentations [1].

Among the most common autoimmune disorders that befell humanity are “systemic lupus erythematosus (SLE)” and “rheumatoid arthritis (RA)” [1]. SLE is characterized by autoantibodies directed against nuclear and cytoplasmic antigens, manifesting as a remitting and relapsing course of disease, presenting with multiorgan system involvement and sometimes with isolated organ or organ system involvement [3]. A wide range of clinical manifestations of SLE involves commonly renal, dermatological, neuropsychiatric, and cardiovascular symptoms. The laboratory biomarkers used to effectively diagnose and monitor SLE activity include antinuclear antibodies (ANA), anti-double stranded DNA antibodies (anti-dsDNA antibodies), anti-Smith antibodies (anti-Sm antibodies), and low levels of complement 3 and 4 levels [4]. It is now an important social and public health problem since the medication and multidisciplinary approach to treating SLE can only control symptoms and delay progression but cannot cure it completely [4]. RA is another common autoimmune disease that is characterized by a chronic inflammatory process dominantly affecting the joints along with extra-articular organs including the heart, kidney, lung, digestive system, eye, skin, and nervous system [5]. The laboratory biomarkers used to diagnose and monitor disease activity include rheumatoid factor (RA factor) and anti-cyclic citrullinated peptide (anti-CCP) antibodies [5]. The clinical status of RA patients has significantly improved over the years owing to medical advancements, especially promising results with disease-modifying anti-rheumatic agents (DMARDs). Unfortunately, we have yet to discover a means to completely cure the disease [5].

Among various immunological dysregulations that lead to the pathogenesis of autoimmune diseases, regulatory T cell (Treg) deficiency, reduction, instability, reduced vitality, and dysfunction are seen across multiple autoimmune diseases including SLE and RA [6]. Since many studies on the regenerative function of Treg cells have been published, the conclusion derived from these studies was that the ideal treatment strategy would be to induce self-tolerance before tissue damage occurs by restoring Treg cell population and their function [7-10]. Interleukin 2 (IL2) is a key cytokine for T cell activation and proliferation, and since natural Treg cells express high levels of CD25 (IL2 receptor alpha chain), they are highly sensitive to stimulation via IL2 [10]. This rationale led to various studies demonstrating the restorative effects of low-dose IL2 therapy leading to the expansion of the Treg cell population, attenuation of other immune mediators, and improvement of disease activity in patients suffering from various autoimmune disorders, the phenomenon referred to as immunomodulation [11-13].

We now know that the pathophysiology of various autoimmune diseases shows a greater degree of similarities and low-dose IL2 shows promising results in autoimmune diseases in general and, in various studies, is found to have a better safety profile as compared to standard therapy alone. However, since different autoimmune diseases show variability in their pathophysiological processes leading to different biological markers and clinical manifestations in each disease, it would be credible to assume that low dose IL2 may not entirely have the same effect on the improvement of biological markers and clinical disease activity in different autoimmune disorders. This study aims to compare the efficacy of low-dose IL2 therapy in patients suffering from SLE versus patients suffering from RA.

## Review

Methods

We conducted this systematic review using Preferred Reporting Items for Systematic Reviews and Meta-Analyses (PRISMA) guidelines [14].

Search Strategy

We used the following databases to conduct our research: PubMed, PubMed Central (PMC), Medical Literature Analysis and Retrieval System Online (MEDLINE), and Google Scholar. We researched the databases on 12th September 2023. The summary of the keywords used and the PubMed search builders employed for this study is demonstrated in Table 1.

**Table 1:** Keywords and PubMed search builders SLE, systemic lupus erythematosus; RA, rheumatoid arthritis; IL2, interleukin 2

Concept	Keywords	PubMed Search Builder
Systemic Lupus Erythematosus	SLE, Systemic Lupus Erythematosus	SLE OR Systemic Lupus Erythematosus OR ( "Lupus Erythematosus, Systemic/drug therapy"[Majr:NoExp] OR "Lupus Erythematosus, Systemic/immunology"[Majr:NoExp] OR "Lupus Erythematosus, Systemic/therapy"[Majr:NoExp] )
Rheumatoid Arthritis	RA, Rheumatoid Arthritis	RA OR Rheumatoid Arthritis OR ( "Arthritis, Rheumatoid/drug therapy"[Majr:NoExp] OR "Arthritis, Rheumatoid/immunology"[Majr:NoExp] OR "Arthritis, Rheumatoid/therapy"[Majr:NoExp] )
Low Dose Interleukin 2 Therapy	Low Dose Interleukin 2 Therapy, Low Dose IL2 Therapy, IL2 Therapy, Immunomodulation	Low Dose Interleukin 2 Therapy OR Low Dose IL2 Therapy OR IL2 Therapy OR Immunomodulation OR ("Immunomodulation/drug effects"[Majr:NoExp])

The final search strategy employed on all four databases is summarized in Table 2.

**Table 2:** Final search strategy on all databases SLE, systemic lupus erythematosus; RA, rheumatoid arthritis; IL2, interleukin 2

Databases	Final Search Strategy
PubMed/ PubMed Central/ MEDLINE	Systemic Lupus Erythematosus OR SLE OR ( "Lupus Erythematosus, Systemic/drug therapy"[Majr:NoExp] OR "Lupus Erythematosus, Systemic/immunology"[Majr:NoExp] OR "Lupus Erythematosus, Systemic/therapy"[Majr:NoExp] ) AND RA OR Rheumatoid Arthritis OR ( "Arthritis, Rheumatoid/drug therapy"[Majr:NoExp] OR "Arthritis, Rheumatoid/immunology"[Majr:NoExp] OR "Arthritis, Rheumatoid/therapy"[Majr:NoExp] ) AND Low Dose Interleukin 2 Therapy OR Low Dose IL2 Therapy OR IL2 Therapy OR immunomodulation OR ("Immunomodulation/drug effects"[Majr:NoExp])
Google Scholar	Systemic Lupus Erythematosus OR SLE AND Rheumatoid Arthritis AND Low Dose Interleukin 2 Therapy OR Low Dose IL2 Therapy OR IL2 Therapy

Inclusion and Exclusion Criteria

Inclusion criteria for this review included studies that were randomized controlled trials (RCTs), clinical trials, and observational studies strictly relevant to the topic, were peer-reviewed, in English language, containing free full texts, and were no more than 10 years old. Animal studies and all grey literature were excluded from this review.

Screening of Articles

After obtaining the articles from the search strategies employed as shown above, automation tools were applied and irrelevant articles were removed from the search. These automation tools applied were according to the inclusion criteria of this study. Then the articles retrieved were screened based on their titles and abstracts. Finally, the articles were short-listed and subjected to quality assessment.

Quality Appraisal Tools

We conducted the quality appraisal of the final seven articles. The articles satisfying 70% of the appraisal parameters were included in the systematic review.

Results

Search Outcome

**Figure 1:**
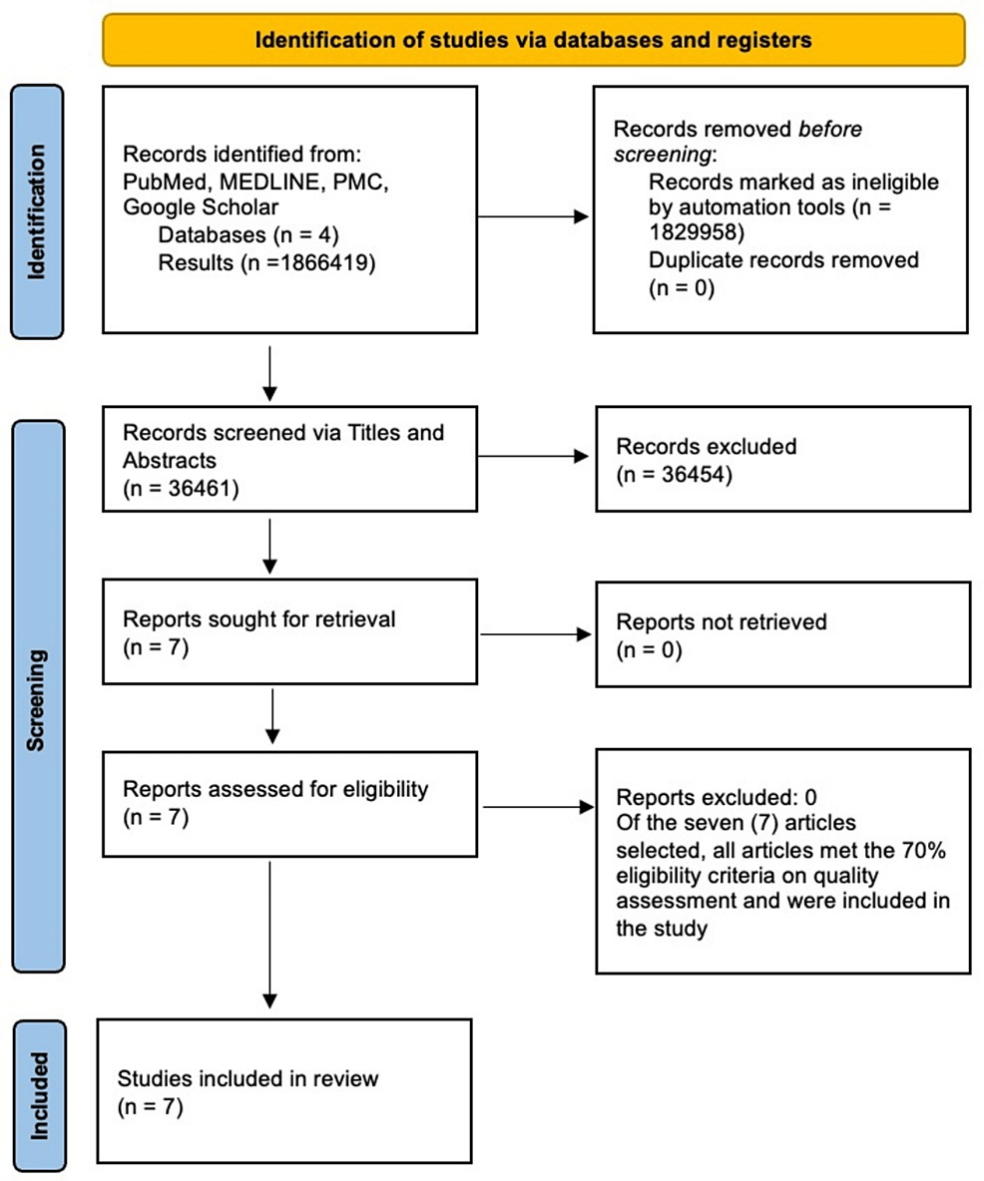
PRISMA flow diagram demonstrating article selection process PRISMA, Preferred Reporting Items for Systematic Reviews and Meta-Analyses; MEDLINE, Medical Literature Analysis and Retrieval System Online, PMC: PubMed Central

The literature search for this systematic review yielded a total of 1866419 studies from all four databases. After the eligibility criteria filters were applied, 36461 articles remained. These 36461 articles were subsequently screened and 36454 articles were removed based on their titles/abstracts. The remaining seven articles were subjected to quality assessment using the relevant quality assessment tools. All seven articles that were subjected to quality assessment met the standards of quality appraisal and were included in the review. Figure 1 demonstrates the search outcome in the form of a PRISMA flow diagram.

Quality Appraisal Outcome

Table 3 demonstrates the quality appraisal outcome of selected studies.

**Table 3:** Quality appraisal outcome

Study	Type of Study	Quality Appraisal Tool	Quality Appraisal Score
He et al., 2020 [15]	Randomized Controlled Trial	Cochrane Risk-of-Bias (RoB) Assessment Tool	91%
Zhao et al., 2019 [16]	Non-randomized Clinical Trial	Newcastle-Ottawa Scale (NOS)	88%
Zhang et al., 2022 [17]	Non-randomized Clinical Trial	Newcastle-Ottawa Scale (NOS)	88%
Humrich et al., 2019 [18]	Non-randomized Clinical Trial	JBI Critical Appraisal Tool	90%
Zhang et al., 2022 [19]	Randomized Controlled Trial	Cochrane Risk-of-Bias (RoB) Assessment Tool	83%
Wang et al., 2022 [20]	Randomized Controlled Trial	Cochrane Risk-of-Bias (RoB) Assessment Tool	75%
Zhang et al., 2021 [21]	Non-randomized Clinical Trial	Newcastle-Ottawa Scale (NOS)	100%

Actual Results

Table 4 demonstrates the individual characteristics of selected studies.

**Table 4:** Characteristics and conclusions of articles included in this systematic review IL2, interleukin 2; SLE, systemic lupus erythematosus; SRI, SLE responder index; anti-dsDNA, anti double stranded deoxyribonucleic acid; Th17 cells, T helper 17 cells; Treg cells, regulatory T cells; SLEDAI, systemic lupus erythematosus disease activity index; SELENA, safety of estrogens in lupus national assessment; RA, rheumatoid arthritis; MTX, methotrexate; CDAI, clinical disease activity score; SDAI, simplified disease activity score; ACR, American college of rheumatology; DAS28, disease activity score in 28 joints with erythrocyte sedimentation score; TJC, tender joint count; SJC, swollen joint count

Author and Year of Publication	Type of Study	Purpose of Study	Number of Participants	Results	Conclusion
He et al., 2020 [15]	Randomized Controlled Trial	To formally evaluate the safety and efficacy of low-dose IL2 therapy in patients with SLE and determine whether low-dose IL2 treatment benefits by inducing clinical improvement without increasing incidence of infection.	60 patients in total. 30 received low-dose IL2 with standard therapy and 30 patients received placebo with standard therapy.	At week 24, the SRI-4 response rate in the low-dose IL2 and placebo group was 65.52% and 36.67% (p=0.027), respectively, the clinical remission rate was higher in the IL2 group than placebo and anti-dsDNA antibody titers decreased in the IL2 group and not in the placebo group.	A more rapid disease improvement was observed in the IL2 group. Collectively study provided evidence that low-dose IL2 coupled with standard therapy might be effective and well tolerated in patients with SLE compared to standard therapy alone.
Zhao et al., 2019 [16]	Non-randomized Clinical Trial	To analyze the effect of low-dose IL2 therapy plus rapamycin on the number of Treg, Th17, and ratio of Th17/Treg cells, along with evaluation of therapeutic efficacy of this treatment modality in refractory SLE patients.	50 patients received low-dose IL2 combined with rapamycin. 70 healthy controls were enrolled.	At 24 weeks (compared to 0 weeks), low-dose IL2 and rapamycin increased the absolute number of Treg cells (p=0.002). Although the absolute number of Th17 cells was unchanged (p=0.107), Th17/Treg ratio decreased significantly post-treatment (p=0.007). SLEDAI score was significantly lower (p<0.001), and prednisolone dosage significantly decreased (p<0.001).	This study has confirmed that the imbalance between Th17 cells and Treg cells caused by the significant reduction of Treg cells may be one of the major causes of SLE and modulating this balance via low-dose IL2 combined with rapamycin is a potential approach for treating SLE since it led to improvements in disease activity and reduction in prednisolone dosage in refractory SLE patients.
Zhang et al., 2022 [17]	Non-randomized Clinical Trial	To investigate the alteration and absolute concentrations of lymphocyte subpopulations in SLE patients with different infections and their responses to low-dose IL2 therapy.	495 patients in total. 54 patients received low-dose IL2 therapy. 132 healthy controls were enrolled.	After five consecutive days of low-dose IL2 therapy, significant increases in lymphocyte subpopulations were seen in SLE patients with infection as effectively as seen in SLE patients without infection: T (p<0.001), B (p<0.001), CD8+ T (p<0.01), NK (p<0.001), Th1 (p<0.01), Treg (p<0.10). This increase in lymphocyte cell subpopulations was comparable to levels in healthy controls.	Patients with SLE had disturbance of the immune system by a decreased number of various lymphocyte subsets. Especially those suffering from infections. Preliminary findings suggest that the low absolute number of these cells may be an indicator of possible infection in SLE patients and low-dose IL2 therapy proves to enhance the ability to resist infection in SLE patients by restoring a decreased number of lymphocyte subpopulations.
Humrich et al., 2019 [18]	Non-randomized Clinical Trial	To investigate the efficacy, safety, tolerability, and immunological effects of low-dose IL2 therapy in moderately to severely active SLE patients.	12 patients who received low-dose IL2 therapy were enrolled in this study.	Low-dose IL2 therapy was well tolerated at 0.75-1.5 million IU. 8 of 12 patients received 3 million IU doses of low-dose IL2 during 2^nd^ treatment cycle which showed increased frequency and severity of adverse effects. At day 62, 11 of 12 patients met the primary endpoint with the proportion of CD25hi-expressing cells among CD3+CD4+FOXP3+CD127lo Treg cells doubling (p=0.0005). 10 of 12 patients had a reduction of SELENA-SLEDAI scores with a median score of 5.0 compared with a baseline score of 10.0 (p<0.05). Transient but significant increases in C3 factor were noted (p=0.0078) but no significant reduction in concentrations of anti-dsDNA antibody titers was seen (p=0.8501).	The provision of low-dose IL2 therapy is well tolerated and results in increased proportions of total and CD25-expressing Treg cells. Increases in the Treg cell population were significant but transient which suggests that cyclical treatment might not be optimal for sustained response. Low-dose IL2 was clinically efficacious in 8 of 12 patients and was sustained throughout the treatment cycles in this study. However median daily prednisolone dose did not change significantly between the start and end of the study.
Zhang et al., 2022 [19]	Randomized Controlled Trial	To evaluate the potential effects and safety of low-dose IL2 therapy along with methotrexate (MTX) on Rheumatoid Arthritis (RA) and identification of factors in predicting potential responses to the treatment of low-dose IL2.	47 patients in total. 23 patients received low-dose IL2 with MTX and 24 patients received placebo with MTX.	At week 24 ACR-20, ACR-50, and ACR-70 response in low-dose IL2 + MTX group compared to placebo + MTX group was 76.5% vs 56.5% (p=0.08), 58.8% vs 34.8% (statistically insignificant) and 23.5% vs 8.8% (statistically insignificant) respectively. Improvements from baseline CDAI and SDAI indices were significantly greater across time points for the low-dose IL2 + MTX group than the placebo + MTX group (p=0.018 and p=0.015 respectively). DAS28-ESR score <2.6 was achieved by 35.3% of patients in the low-dose IL2 + MTX group as compared to 26.1% in the placebo + MTX group (statistically insignificant). The adverse effect rate in the low-dose IL2 + MTX group was 47.8% as compared to 37.5% in the placebo + MTX group but the incidence of severe adverse effects through week 24 was low and similar between the two groups.	Low-dose IL2 further increased the efficacy of MTX with significant improvement of CDAI, SDAI, and ACR responses, and the treatment was well tolerated. In accordance with clinical improvements, inflammatory cytokines were dampened with low-dose IL2 therapy. Increased Treg cell population and attenuation of Th17 cell percentage such that the Treg/Th17 ratio increased significantly.
Wang et al., 2022 [20]	Randomized Controlled Trial	To compare the status of peripheral blood lymphocyte and CD4+ T cell subsets of refractory RA with healthy donors and whether low-dose IL2 could effectively induce remission of refractory RA by upregulating Treg cells.	41 total patients and 40 healthy individuals. Of 41 patients, 15 patients received conventional treatment and 26 received low-dose IL2 therapy in conjunction with conventional treatment.	Before treatment, there was seen to be no difference between Th17 cells between RA and healthy individuals (9.0 vs 7.4, p = 0.132) but the Treg cell population was significantly lower in RA patients as compared to the healthy group (19.5 vs 35.5, p < 0.001). Following treatment, the low-dose IL2 group showed significant increases, as compared to the conventional therapy group, in T cells (1227 vs 990 cells/µl, p = 0.015), in CD4+ T cells (823 vs 564 cells/µl, p = 0.003) and Treg cells (65 vs 23 cells/µl, p < 0.001). In the low-dose IL2 group vs conventional therapy group, a significant decrease in DAS28 score (3.60 ± 0.96 vs 2.85 ± 0.67, p = 0.005), 28 tender joint count (3.73 ± 2.79 vs 0.94 ± 1.00, p = 0.001) and swollen joint count (1.40 ± 1.54 vs 0.42 ± 0.70, p = 0.011) were seen. There were no severe adverse effects observed with low-dose IL2 therapy except for mild reactions at the site of injection in 2 of 26 low-dose IL2 therapy group patients.	Low-dose IL2 therapy increased Th17 cells slightly while a significant increase in Treg cells was seen along with other peripheral blood lymphocytes. This increase was correlated with relieving patients' clinical symptoms without expected adverse effects. Hence low-dose IL2 therapy can induce remission of RA disease activity rapidly in refractory RA patients.
Zhang et al., 2021 [21]	Non-randomized Clinical Trial	To measure the absolute counts of CD4+ T cell subsets in patients with RA and healthy controls and to elucidate the immune mechanism of RA.	888 patients with RA and 100 healthy controls. Of 888 patients, 233 received low-dose IL2 and the rest received conventional therapy.	Treatment indicators of disease activity before and after low-dose IL2 therapy showed a significant decrease; DAS28 (5.44 ± 1.34 vs 3.66 ± 1.09, p < 0.001), TJC (10.07 ± 7.97 vs 3.94 ± 4.76, p < 0.001) and SJC (6.40 ± 7.63 vs 1.70 ± 3.32, p < 0.001). Patients with low-dose IL2 therapy showed a three-fold increase in absolute counts of Treg (26.26 ± 15.51 to 92.80 ± 64.92) and a two-fold increase in CD4+ T cells and its subsets (p < 0.001). Th17/Treg ratio decreased significantly to the level of healthy controls. Low-dose IL2 was tolerated in all patients with RA.	Decreased absolute counts of circulating CD4+ T cell subsets were observed in patients with RA and circulating Tregs which mediate immune tolerance, may be involved in the pathogenesis of RA. Tregs can be restored by low-dose IL2 therapy without obvious side effects and effectively induce remission in RA patients.

Discussion

Autoimmune diseases have an overall prevalence of 3-5% in the general population and although considered a rare group of diseases, prevail almost indefinitely through the lives of its victims [1]. Over the course of many years, various advances have been made discussing different pathophysiological bases of these diseases and developing treatment approaches for remission or optimal control of these diseases. Contemporary treatment modalities for optimal disease control or remission of SLE and RA include corticosteroids, biologic agents, and immunosuppressants. These treatment modalities, however, offer incompletely effective outcomes which are further offset by significant side effects of the drugs [22-24]. This warrants a search for a treatment modality that offers better clinical outcomes without associated adverse effects. It led to a novel treatment approach that encompasses using low-dose IL2 to rectify immunological imbalances leading to the SLE and RA and, hence, achieving remission with minimal risk of treatment-associated adverse effects. This study focuses on the application of novel low-dose IL2 therapy in these two autoimmune diseases i.e. SLE and RA, as well as the comparability of these new interventions’ efficacy and safety among patients of SLE and RA.

Biomarker Improvement and Clinical Efficacy of Low-Dose IL2 in SLE vs RA

He et al. demonstrated in the randomized clinical trial that the group of SLE patients in which low-dose IL2 therapy, along with conventional therapy, was employed for the treatment of SLE offered a higher response rate, which was statistically significant, as compared with the placebo group in which only conventional therapy was used [15]. The SRI-4 index at the end of the study (i.e. week 24) was 65.52% in the low-dose IL2 group vs only 36.67% in the placebo group [15]. Significantly more reductions in SELENA-SLEDAI scores were observed in the low-dose IL2 group [15]. The complete remission of lupus nephritis was also significantly higher in the low-dose IL2 group versus the placebo group at week 24 (53.85% vs 16.67%; p = 0.036) [15]. The clinical remission in both the low-dose IL2 group and placebo group was accompanied by tapering of the corticosteroid dose; however, the phenomenon was more pronounced in the low-dose IL2 group [15]. At week 24, 44.83% (13/29) of patients in the low-dose IL2 group had reduced prednisone dose by more than 50% as compared to 33.33% (10/30) in the placebo group [15]. This study also showed that low-dose IL2 therapy led to a significant decrease in anti-nuclear antibodies (ANA) and anti-double-stranded DNA antibodies over the course of 24 weeks as compared to patients treated with only conventional therapy [15]. Zhao et al. evaluated the clinical efficacy of low-dose IL2 therapy in conjunction with rapamycin in their study using the SLEDAI score only [16]. They observed decreased SLEDAI scores of SLE patients in the 24th week as compared to the 0 week and the decrease was statistically significant [16]. The SLEDAI score at week 24 was 3.54 ± 2.37 which suggested that the disease is at the inactive stage [16]. A reduction in the dosage of prednisolone was observed in refractory SLE patients in this study [16]. This development reinforces the conclusive evidence provided by He et al. that low-dose IL2 therapy is more efficacious than conventional therapy alone, which is reflected in the fact that corticosteroid dosage required for disease remission in patients treated with low-dose IL2 therapy is low as compared to conventional therapy alone [15,16]. Zhao et al. also demonstrated that a slight increase in complement factor C3 and C4 levels was seen but was statistically insignificant [16]. Humrich et al. demonstrated in their clinical trial that SLE patients treated with low-dose IL2 showed a robust response to the therapy [18]. Patients studied in this trial had inadequately controlled disease by at least two different previous conventional immunosuppressive therapies. Eighty-three percent of patients had a reduction of the SELENA-SLEDAI score at day 62 with a median score of 5.0 as compared with a median score of 10.0 at the start of the study [18]. Sixty-seven percent of patients achieved a clinical response with a complete disappearance of active clinical manifestations like arthritis, rash, alopecia, mucosal ulcers, and myositis [18]. All these responders showed a significant decrease in the SELENA-SLEDAI score before day 14 and in 70% of these responders, the median reduction in the SELENA-SLEDAI score was maintained until day 83 of the study [18]. This study also demonstrated a transient but significant improvement in complement factor C3 noted after each cycle and at the end of the study i.e. day 62 [18]. A significant increase in complement factor C4 concentration was also seen after the first treatment cycle but no significant change in anti-double-stranded DNA antibodies and other disease-associated autoantibodies was seen [18]. The facts investigated and proved, as stated above, showed that low-dose IL2 shows promise as a treatment modality for SLE patients; however, it was seen in this study that the immunomodulation induced by low-dose IL2 was transient [18]. It, thus, suggested that the cyclical treatment regime employed in this study may not be optimal for sustained immunomodulation and, therefore, response to treatment [18].

Current treatment regimens for RA mainly rely on conventional synthetic disease-modifying anti-rheumatic drugs (DMARDs) and biologic agents which are associated with substantial adverse effects including hepatic damage, cytopenia, and various infections. Methotrexate (MTX) is the most widely accepted first-line therapy but is not efficacious in a large population of patients and potential adverse effects limit its use. Zhang et al. demonstrated that RA patients treated with low-dose IL2 in conjunction with MTX had a better clinical outcome as compared to placebo with MTX treatment [19]. This was evident from measuring the indices for disease activity of RA i.e. SDAI and CDAI and ACR response indices [19]. The low-dose IL2 group had significantly greater improvements in CDAI and SDAI over the course of the study [19]. ACR20, ACR50, and ACR70 also showed a greater response to treatment in the low-dose IL2 group as compared to placebo [19]. This study showed a direct relation between this significant improvement in disease activity scores of RA with the immunomodulation that is brought about by low-dose IL2 therapy [19]. It was also demonstrated that during the course of their study, there was no significant decrease in rheumatoid factor (RF) and anti-cyclic-citrullinated peptide (anti-CCP) [19]. Wang et al. demonstrated that patients who received low-dose IL2 therapy in conjunction with conventional therapy had a greater clinical response with a significant decrease in the DAS28 score, 28 tender count, and swollen joint count as compared with RA patients being treated with only conventional therapy [20]. The findings in this study consolidated the findings of the study carried out by Zhang et al., which suggested that the significant improvement seen in the low-dose IL2 group is directly correlated with immunomodulation. Similarly, Zhang et al. demonstrated that low-dose IL2 therapy led to greatly decreased indicators of disease activity i.e. DAS28, TJC, SJC, and CRP, as compared to baseline values and the results were statistically significant [21]. The study carried out by Zhang et al. mainly focused on the pathophysiological basis of greater improvement in the low-dose IL2 therapy group as compared to the conventional therapy group alone [21]. It successfully demonstrated that the greater degree of improvement was a direct result of immunomodulation leading to immune tolerance and thus, effectively equipping the human body to tackle RA [21].

It is observed through this review that low-dose IL2 therapy in conjunction with respective standard therapies has comparable benefits in terms of disease remission in both SLE and RA. There was significant improvement in the respective disease activity scores of SLE and RA, and this phenomenon was strongly suggestive of the fact that immunomodulation inducing immune tolerance may be a superior treatment modality, as compared with contemporary practices, employed for the treatment of SLE as well as RA. While it is true that the disease activity of both SLE and RA respond comparably to low-dose IL2 therapy, the biomarkers do not seem to respond equally. Treatment with low-dose IL2 in SLE has shown to be beneficial in restoring the complement factors as well as decreasing the autoantibodies implicated in the disease, while the same could not be said about RA patients being treated with low-dose IL2 therapy. One reason for this discrepancy could be bias due to limited literature on the effects of low-dose IL2 therapy on RA biomarkers.

Safety of Low-Dose IL2 in SLE vs RA

A randomized clinical trial by He et al. demonstrated that the use of low-dose IL2 therapy along with immunosuppressant and biologic therapies already being employed for disease control offered a more effective and rapid disease control with a lower incidence of adverse effects [15]. Low-dose IL2 therapy caused significantly increased expansion of natural killer (NK) cells which implicated potential augmentation of anti-infectious cellular immunity and possibly explained the lower incidence of treatment-associated severe infections in the patients [15]. Zhao et al. also demonstrated in the non-randomized clinical trials on patients with severe refractory SLE that require large doses of prednisone or cytotoxic drugs, that combination with low-dose IL2 may not only alleviate the disease effectively but also decrease the dose of prednisone and cytotoxic drugs required to induce and maintain remission and ultimately be beneficial to patients in terms of decreased incidence of treatment-associated adverse effects [16]. Zhang et al. conducted a non-randomized clinical trial involving SLE patients with infection who were already taking high doses of prednisone (30mg/day median) and demonstrated a significant increase in Treg cell and CD8+ T cell populations in patients who were given low-dose IL2 therapy [17]. This selective expansion of immune regulatory cells showed promise in studies conducted by Zhao et al. and He et al., which was premised on the rationale behind the expected lower incidence of adverse effects among these individuals. However, this study was conducted over a course of only five days of observation; hence, it was not enough to estimate the direct effect of low-dose IL2 on the treatment of infection, and whether low-dose IL2 therapy could reduce infection rates needed further studies [17]. Humrich et al. also conducted a non-randomized clinical trial involving patients with moderately to severely active SLE. Humrich and his team noted that a range of adverse effects were noted among patients who received low-dose IL2 therapy and five severe life-threatening adverse effects/infections were seen during the follow-up period of the study [18]. Only one of these five adverse effects, i.e. peripheral ischemia, was judged to be treatment-related. [18] Mild to moderate adverse effects, that were noted, disappeared within the few days during the washout periods [18].

Zhang et al. conducted a randomized clinical trial that demonstrated a comparable incidence of adverse effects among the two groups through week 24 of the study [19]. Mild adverse effects, including transient fever and injection site reactions, were observed in both study groups [19]. Severe adverse effects in the study were noted to be singular events and no specific association was seen with treatment [19]. Wang et al. also conducted a randomized controlled trial in which they demonstrated that other than mild reactions at the injection sites in some patients treated with low-dose IL2, no other adverse effects were noted [20]. Similarly, Zhang et al. conducted a non-randomized clinical trial where RA patients were given low-dose IL2 therapy in conjunction with conventional therapy and were followed after five days of treatment regimen [21]. Among these patients, low-dose IL2 was well tolerated and none of them displayed severe adverse effects. Mild adverse effects were observed and included skin rashes over injection sites that healed spontaneously [21].

It can be concluded through this review that low-dose IL2 therapy is a safe treatment modality for patients with SLE or RA; not just because of the low risk of serious adverse effects of the treatment itself, but also because this therapy induces immune tolerance by correcting the immune dysregulation that led to the disease in the first place. This is how it decreases the treatment dosage of conventional therapy drugs the patients are already taking and thus mitigates the adverse effects previously seen in patients treated with conventional therapy alone.

Limitations

This systematic review has certain limitations; one such limitation was that there were differences between variable parameters of individual studies (for example, number of participants, and study duration) included in this review. For example, Zhang et al. conducted a study where 233 patients received low-dose IL2 therapy while Wang et al. conducted a study where only 26 patients received low-dose IL2 therapy. The individual limitation of each study regarding these variable parameters made it difficult to facilitate adequate comparisons. The other most potentially impactful limitation was the scarcity of studies done to evaluate the efficacy and safety of low-dose IL2 therapy, especially in RA. Thus, the efficacy of low-dose IL2 therapy in RA, particularly RA biomarker attenuation, could not be properly compared with SLE biomarker attenuation.

## Conclusions

The systematic review aimed to determine whether low-dose IL2 therapy, along with respective conservative therapy, has a similar safety profile and beneficial effects in patients with RA and SLE, or not. Based on the results gathered from the articles included in this study, there seems to be a comparable safety profile and therapeutic effects of low-dose IL2 therapy on patients from both disease groups. The biomarkers, however, do not seem to respond similarly to low-dose IL2 therapy in both disease groups. The findings of this review suggested that biomarker improvement and attenuation by low-dose IL2 therapy in patients with SLE was consistently evident, while the same effect was not observed for RA patients treated with low-dose IL2 therapy; however, this may be due to limited articles that study the effects of low-dose IL2 therapy on RA biomarkers. Nonetheless, it can be concluded that low-dose IL2 therapy in conjunction with respective conservative therapy has proved to be a superior treatment modality for both SLE and RA, has limited adverse effects as compared to conventional therapy alone, and has a similar therapeutic application and safety profile in both autoimmune diseases.

## References

[1] Wang L, Wang FS, Gershwin ME (2015). Human autoimmune diseases: a comprehensive update. J Intern Med.

[2] Rahman P, Inman RD, El-Gabalawy H, Krause DO (2010). Pathophysiology and pathogenesis of immune-mediated inflammatory diseases: commonalities and differences. J Rheumatol Suppl.

[3] Fortuna G, Brennan MT (2013). Systemic lupus erythematosus: epidemiology, pathophysiology, manifestations, and management. Dent Clin North Am.

[4] Yu H, Nagafuchi Y, Fujio K (2021). Clinical and immunological biomarkers for systemic lupus erythematosus. Biomolecules.

[5] Radu AF, Bungau SG (2021). Management of rheumatoid arthritis: an overview. Cells.

[6] Zhang X, Olsen N, Zheng SG (2020). The progress and prospect of regulatory T cells in autoimmune diseases. J Autoimmun.

[7] Gonzalez-Rey E, Delgado M (2007). Vasoactive intestinal peptide and regulatory T-cell induction: a new mechanism and therapeutic potential for immune homeostasis. Trends Mol Med.

[8] Zhou X, Kong N, Zou H, Brand D, Li X, Liu Z, Zheng SG (2011). Therapeutic potential of TGF-β-induced CD4(+) Foxp3(+) regulatory T cells in autoimmune diseases. Autoimmunity.

[9] Jiang Q, Yang G, Liu Q, Wang S, Cui D (2021). Function and role of regulatory T cells in rheumatoid arthritis. Front Immunol.

[10] Mosanya CH, Isaacs JD (2019). Tolerising cellular therapies: what is their promise for autoimmune disease?. Ann Rheum Dis.

[11] Rosenzwajg M, Churlaud G, Mallone R (2015). Low-dose interleukin-2 fosters a dose-dependent regulatory T cell tuned milieu in T1D patients. J Autoimmun.

[12] He J, Zhang X, Wei Y (2016). Low-dose interleukin-2 treatment selectively modulates CD4(+) T cell subsets in patients with systemic lupus erythematosus. Nat Med.

[13] Al Tabaa O, Hamroun S, Leplay C, Fain O, Klatzmann D, Mekinian A (2022). Cohort study of off-label use of low-dose IL-2 therapy for systemic autoimmune diseases. Clin Exp Rheumatol.

[14] Page MJ, McKenzie JE, Bossuyt PM (2021). The PRISMA 2020 statement: an updated guideline for reporting systematic reviews. BMJ.

[15] He J, Zhang R, Shao M (2020). Efficacy and safety of low-dose IL-2 in the treatment of systemic lupus erythematosus: a randomised, double-blind, placebo-controlled trial. Ann Rheum Dis.

[16] Zhao C, Chu Y, Liang Z (2019). Low dose of IL-2 combined with rapamycin restores and maintains the long-term balance of Th17/Treg cells in refractory SLE patients. BMC Immunol.

[17] Zhang JQ, Zhang SX, Wang J (2022). Low-dose IL-2 therapy limits the reduction in absolute numbers of peripheral lymphocytes in systemic lupus erythematosus patients with infection. Curr Med Res Opin.

[18] Humrich JY, von Spee-Mayer C, Siegert E (2019). Low-dose interleukin-2 therapy in refractory systemic lupus erythematosus: an investigator-initiated, single-centre phase 1 and 2a clinical trial. Lancet Rheumatol.

[19] Zhang X, Miao M, Zhang R (2022). Efficacy and safety of low-dose interleukin-2 in combination with methotrexate in patients with active rheumatoid arthritis: a randomized, double-blind, placebo-controlled phase 2 trial. Signal Transduct Target Ther.

[20] Wang J, Zhang SX, Chang JS (2022). Low-dose IL-2 improved clinical symptoms by restoring reduced regulatory T cells in patients with refractory rheumatoid arthritis: a randomized controlled trial. Front Immunol.

[21] Zhang SX, Wang J, Wang CH (2021). Low-dose IL-2 therapy limits the reduction in absolute numbers of circulating regulatory T cells in rheumatoid arthritis. Ther Adv Musculoskelet Dis.

[22] Kang I, Park SH (2003). Infectious complications in SLE after immunosuppressive therapies. Curr Opin Rheumatol.

[23] Goldblatt F, Chambers S, Rahman A, Isenberg DA (2009). Serious infections in British patients with systemic lupus erythematosus: hospitalisations and mortality. Lupus.

[24] Costello R, David T, Jani M (2019). Impact of adverse events associated with medications in the treatment and prevention of rheumatoid arthritis. Clin Ther.

